# Reliability of the standard goniometry and diagrammatic recording of finger joint angles: a comparative study with healthy subjects and non-professional raters

**DOI:** 10.1186/1471-2474-14-17

**Published:** 2013-01-09

**Authors:** Valdas Macionis

**Affiliations:** 1 Clinic of Rheumatology, Orthopaedics and Traumatology, and Reconstructive Surgery, Vilnius University Faculty of Medicine, Vilnius, Lithuania; 2 Department of Plastic and Reconstructive Surgery, Centre of Plastic and Reconstructive Surgery, Vilnius University Hospital Santariskiu Klinikos, Vilnius, Lithuania

## Abstract

**Background:**

Diagrammatic recording of finger joint angles by using two criss-crossed paper strips can be a quick substitute to the standard goniometry. As a preliminary step toward clinical validation of the diagrammatic technique, the current study employed healthy subjects and non-professional raters to explore whether reliability estimates of the diagrammatic goniometry are comparable with those of the standard procedure.

**Methods:**

The study included two procedurally different parts, which were replicated by assigning 24 medical students to act interchangeably as 12 subjects and 12 raters. A larger component of the study was designed to compare goniometers side-by-side in measurement of finger joint angles varying from subject to subject. In the rest of the study, the instruments were compared by parallel evaluations of joint angles similar for all subjects in a situation of simulated change of joint range of motion over time. The subjects used special guides to position the joints of their left ring finger at varying angles of flexion and extension. The obtained diagrams of joint angles were converted to numerical values by computerized measurements. The statistical approaches included calculation of appropriate intraclass correlation coefficients, standard errors of measurements, proportions of measurement differences of 5 or less degrees, and significant differences between paired observations.

**Results:**

Reliability estimates were similar for both goniometers. Intra-rater and inter-rater intraclass correlation coefficients ranged from 0.69 to 0.93. The corresponding standard errors of measurements ranged from 2.4 to 4.9 degrees. Repeated measurements of a considerable number of raters fell within clinically non-meaningful 5 degrees of each other in proportions comparable with a criterion value of 0.95. Data collected with both instruments could be similarly interpreted in a simulated situation of change of joint range of motion over time.

**Conclusions:**

The paper goniometer and the standard goniometer can be used interchangeably by non-professional raters for evaluation of normal finger joints. The obtained results warrant further research to assess clinical performance of the paper strip technique.

## Background

Graphical presentation of finger range of motion by means of malleable wire tracing is a recognized adjunct to the standard goniometry [1]. This method, however, has been shown to be of inadequate reliability [2]. The range of motion of the finger joints can also be diagrammatically visualized by tracing the arms of an ad hoc goniometer obtained by criss-crossing two folded paper strips [3]. This simple tool can be a quick substitute to the standard goniometer in clinical situations when the latter is unavailable and allows evaluation of finger joint positions, where application of the standard goniometer is impossible (Additional file 1). It has been suggested that the performance of the diagrammatic goniometry should be comparable with that of the standard procedure because both methods are technically similar. As an initial step to test this supposition, the current investigation determined measurement reliability [4,5] for the paper and standard goniometer in non-clinical imitated situations when there was no change in finger range of motion and when the range of motion changed over time.

## Methods

### Rationale of the study design

Search for a possible research model revealed 28 reliability studies involving standard finger goniometry [2,6-37]. A larger part of the studies was carried out on subjects with normal hands [2,6-10,14-16,23,26,29-31,33,35,36]. Taking into account the novelty of the diagrammatic technique and difficulties in carrying out a comparative reliability study of a considerable extent on patients, the current exploration opted for healthy subjects as well. The present investigation chose static finger position model, since in healthy subjects, only a few finger joint postures can be obtained by using standard types of motion [9,12,14,33,36]. Previous researchers ensured stability of the desired finger positions by employing various palmar blocks [7,10,16,29,30,35] and splints [2,15,23,26]. Due to skin mobility and suppleness, however, it seems difficult to achieve steadiness of the joint angle with palmar supports alone. The use of hand cast [24], transarticular pinning of the cadaver finger joints in various degrees of flexion [31], and wooden finger joints [35] is arguably too artificial. Only one study used change of finger motion due to a treatment to test inter-goniometer reliability [6]. However, none of the static models have been employed for this purpose. The current study designed a stabilization system for fingers taking into account the experience and limitations of the previous explorations. Earlier method comparisons, as a rule, involved professional raters who must have been more experienced with one of the techniques under evaluation. Therefore, and considering the extent of the objectives of the present investigation, this study chose to include non-professional evaluators with a similar medical background.

### Ethics statement

The study was approved by Vilnius regional ethics committee for biomedical research. Written informed consents of the participants were obtained before the study.

### Participants and study design

Twenty-four healthy, third- and fourth-year medical students were included in the study in the order of their response to an advertisement inviting to participate in a goniometric reliability study for reimbursement. The key criterion in selecting the participants was similarity of their academic and practical background. None of the participants had considerable experience in goniometry; however, all of them were familiar with the concept of goniometry through their earlier study. The age of the participants ranged from 20 to 24 years. Additionally, 2 fourth-year medical students were invited to participate in a separate reliability study of computerized evaluation of joint angle diagrams.

**Figure 1:**
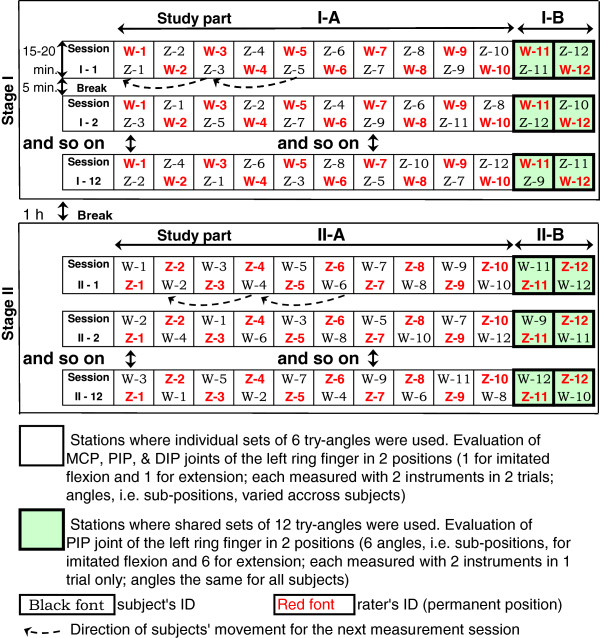
Scheme of the study.

The 24 participants were randomly assigned to the rater or subject group, each including 12 people. The raters were randomly subdivided into subgroups of 10 and 2 to perform different tasks. The study consisted of 2 procedurally identical replicate stages, stage I and stage II. In the study stage II the participants changed their roles (Figure 1, Additional file 2). Each replicate stage of the study included procedurally different parts A and B according to the subdivision of the raters into subgroups of 10 and 2. Thus, the study included replicate parts I-A and II-A with 10 different raters in each and replicate parts I-B and II-B with 2 different raters in each. All raters of the same stage evaluated the same remaining 12 participants acting as subjects under evaluation. The replicate study parts A were designed to compare the goniometers side-by-side in measurement of the metacarpophalangeal (MCP), proximal interphalangeal (PIP), and distal interphalangeal (DIP) joints set at angles varying from subject to subject (Additional file 2). The replicate study parts B were planned to compare the instruments by parallel evaluations of the PIP joint angles similar for all subjects in a situation of simulated change of joint range of motion over time (Additional file 2).

### Equipment

**Figure 2:**
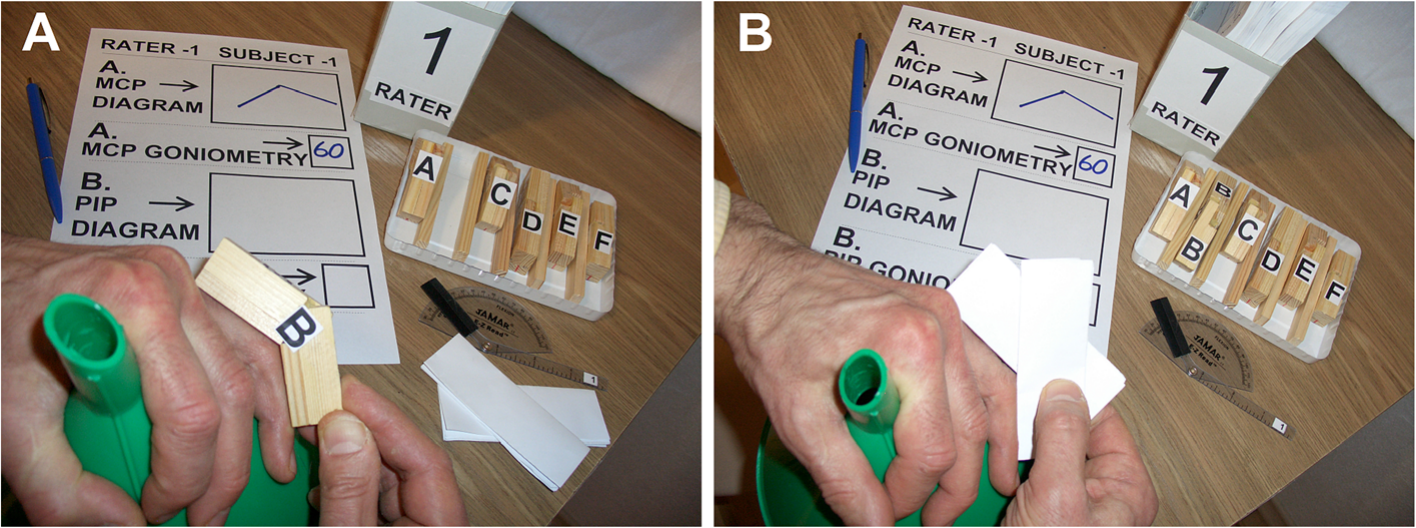
**(A, B) Simplified illustration of the evaluation station and procedure. **(**A**) Stabilization of subject’s hand by grasping a funnel and application of a try-angle to obtain an appropriate position of the PIP joint. (**C**) Evaluation of the obtained joint posture with the paper strip goniometer.

For joint angle measurement, the study employed the improvised paper goniometer (two approximately 10.5 cm by 3.8 cm rectangular paper strips obtained by folding A4 paper sheets) and a standard flexion-hyperextension plastic transparent finger goniometer (Jamar E-Z Read) graduated in 1 degree increments (Figure 2). A plastic cover was used to mask the pointer of the goniometer during the evaluations. The raters entered the measurements into blanks unique for each rater-subject combination pair. Plastic funnels and triangle rulers were used as supports for subjects’ fingers. To set the finger joints in appropriate postures, custom made wooden try-angles (try-square type guides) were applied over the dorsal aspect of the joint (Figure 2a). There were 12 individual sets of 6 try-angles distributed to each subject and 2 shared sets of 12 try-angles to be used by all subjects (Additional file 2). The individual sets contained 3 subsets of 2 try-angles, one pair for each of the finger joints to imitate position of incomplete flexion and extension. Similarly, in the shared sets there were 2 subsets of 6 try-angles, one subset for each of the positions of imitated extension and flexion of the PIP joint only. The angles of the try-angles (or standard angles) were varied to produce different sub-positions of flexion and extension. Importantly, each of the 2 subsets of 6 try-angles in the shared sets enabled 6 different sub-positions of the PIP joint (Additional files 2 and 3).

### Procedures

#### Preparatory procedures

A pilot exploration employing a healthy subject and 17 raters was performed to elucidate possible technical problems of the study.

Two weeks before the study, the participants were sent step-by-step instructions with the appropriate images of the procedure and the equipment. At least a week before the study, the equipment and procedures were demonstrated to the participants live. Example try-angles, triangle rulers, and paper strips were distributed for individual training at home. Taking into account the unusual manipulative task of the diagrammatic goniometry, the participants learned to copy printed angles by using the paper goniometer individually or as participants of another study. Two days before the study, the participants were required to answer a short quiz testing the knowledge of their tasks in the study.

#### Procedures on the day of study

The study was conducted in a spacious auditorium. The raters and subjects faced each other across a long narrow table and sat along the table sides in checkmate pattern. The raters’ locations were permanent, while the subjects, having completed an evaluation session, moved along the table sides clockwise bypassing the neighboring raters to be evaluated by the next rater across the table (Figure 1).

The subjects’ task in all study parts was to stabilize their left ring finger joints in postures set up by grasping a funnel or a triangle ruler and by applying appropriate try-angles over the dorsal aspect of the joint (Figure 2a).

In the replicate study parts A, the subjects used their individual try-angle sets at the 10 evaluation stations (Figure 1, Additional file 2). The values of the angles of individual try-angles were randomly distributed across the finger joints and across the subjects. The angles of the individual try-angles of the same subject were of different magnitude, and none of the subjects had the same combination of the angle magnitudes (Additional file 3). Raters of the study parts A had to obtain twice the MCP, PIP, and DIP joint angles in each of the two positions (flexion and extension) by using both goniometers (Additional file 2) .

In the replicate study parts B, the subjects employed the shared try-angle sets permanently available at the appropriate 2 evaluation stations (Figure 1, Additional file 2). Both shared sets were almost identical in the magnitude of the standard angles; however, the order of the try-angles in the sets was different (Additional file 3). The task of the two raters of the study parts B included only evaluation of the PIP joint in the 6 sub-positions of each of the two positions with both instruments in a single trial (Additional file 2).

When evaluating the joints, the raters were required to do their best to align the instrument arms as close as possible to the position of the anatomical axes of the appropriate bones. Dorsal method of placement was used for both instruments. After aligning the arms of the standard goniometer, the rater removed the cover from the pointer and read the value together with the subject to exclude reading errors; the obtained value was entered into the blank. The angles, obtained by proper alignment of the paper strips, were drawn onto the appropriate sections of the blanks by using edges of the paper strips as rulers. If the arms of the standard goniometer or paper strips were inadvertently displaced during the evaluation, the measurement was repeated.

The procedure protocol also included relaxation of the subject’s hand between the measurements and short breaks between the evaluation sessions. As the length of the evaluation sessions differed from rater to rater, the intervals between sessions also varied. The participants were free to choose longer brakes if they felt tired.

#### Evaluation of diagrams

All the blanks with the recorded angles of the joints were scanned. The scanned diagrams were magnified, and their angles were measured to the nearest degree by the same researcher with ImageJ program. Each diagram was measured at least twice without reference to the previous results. If the results of the two computerized measurements were different, the diagram was remeasured again. If 2 identical measurements were not obtained, mean of the measurements was found and rounded off to the nearest degree. To assess intra-rater and inter-rater reliability of the latter procedure, two invited medical students remeasured 48 randomly chosen scanned diagrams. Computerized evaluation instead of a simple use of a traditional protractor was chosen to equalize varying sizes of the hand drawn diagrams and to avoid errors of hand-done measurements.

### Independence of observations

Although dependency of observations is inherent to within-subject designs, care was taken to ensure the required rater related independence of measurements [5,38]. To prevent any form of communication of the obtained angles, the current study design included checkmate arrangement of the subject and rater pairs, alternating use of the instruments, proceeding to trial 2 only after completing trial 1 for all joints and both instruments, irregular arrangement of the standard angle magnitudes, and masking the pointer of the standard goniometer. Also, the participants were not allowed to share the results of their measurements and were made aware that the standard angles varied widely across the subjects and joints.

### Statistical approaches

#### Measures of reliability

Measurement reliability has been expressed in relative and absolute measures [39,40]. In the current study, the reliability term was used as a hypernym for expressions defining various aspects of measurement uncertainty [39,40], although some authors have used agreement term for this purpose [41] or have understood reliability in a narrower sense [4,5,41].

For continuous variables, the most common measure of relative reliability is intraclass correlation coefficient (ICC) accompanied by appropriate analysis of variance (ANOVA) [40]. Differently from the previous studies, which used the popular models of ICCs described by Shrout and Fleiss [42], the current investigation employed concurrent assessment of reliability proposed by Eliasziw et al. [43]. Unlike calculating the traditional ICCs, the method of concurrent assessment allows simultaneous estimation of intra-rater and inter-rater reliability along with the hypothesis testing in cases when multiple raters evaluate multiple subjects and perform more than one measurement per subject. In respect to the traditional models, the concurrent methodology has been cited as a more advantageous approach [44].

For the clinician, however, reliability coefficients are less important than measures of absolute reliability like the standard error of measurement (SEM), which (when multiplied by 1.96) indicates how far from the hypothetical true value [38,39,45] the measurement obtained by a practitioner could be [40]. The SEM enables derivation of other measures of absolute reliability including the limits of agreement [46] and the minimal detectable change (MDC). The MDC defines the difference that should be obtained between 2 successive measurements on the same subject over time to state that the real change has occurred. In this study the MDC, also referred to as minimal detectable difference [38] or repeatability coefficient [45,47], was found by using formula MDC = SEM x 1.96 x √2 [4,40,45].

Additionally, following a previous suggestion [41], the current study employed intuitive descriptive approaches. To facilitate interpretation of goniometric reliability, proportions of clinically non-meaningful ≤5-degree differences between repeated measurements (here also named “≤ 5-degree agreement”) were analysed [4]. Also, in the smaller B component of this study, mean measurement differences and their standard deviations were employed to reflect absolute reliability [38,46].

#### Sample size estimation

The main attention in this investigation was directed towards calculating intra-rater and inter-rater ICCs and SEMs in the study parts A. The other components of the study were designed as pilot investigations. Balanced numbers of subjects and raters were planned to ensure synchrony of the evaluation sessions. Ten raters were expected to perform 2 repeated measurements (trial 1, trial 2) of the same joint in the same position with the same instrument, which summed up to 20 observations per subject. The ICCs were expected to reach 0.9. However, taking into account the conventionally acceptable lowest ICC values [38], reliabilities of 0.7-0.75 could also be considered as adequate for non-professional raters. Using an earlier proposed formula [48] with the above values and the recommended levels of α=0.05 and β=0.2 resulted in sample sizes between 8 and 12 (Additional file 4).

#### Data Analysis

Each of the replicate study stages was analyzed separately. The significance level was set at p < 0.05.

#### Exploratory data analysis

Exploratory data analysis included obtaining descriptive statistics, searching for outliers, and assessing the normality of distribution of the appropriate data sets by means of Shapiro-Wilk tests and the analysis of histograms and Q-Q plots.

#### Analysis of the study parts A

In the replicate study parts A, 2x2x10 (trial x goniometer x rater) and 2x10 (goniometer x rater) repeated measures ANOVAs were run for each position-joint and trial-position–joint data set, respectively, to assess the main effects and interactions of goniometer, trial, and rater. The sphericity assumption was tested by using Mauchly’s test with appropriate epsilon adjustments.

For concurrent assessment of reliability, the pertinent mean squares were found by running two-factorial univariate ANOVAs [43]. Subject and rater were random effects because the participants were selected randomly and there was no interest in particular raters. Homogeneity of variances was tested with Leven’s test. The necessary variance components were calculated using the obtained mean squares. The intra-rater and inter-rater ICCs, their lower limits of 95% one-sided lower-limit confidence intervals (LLs of 95% one-sided L-L CI), and SEMs were simultaneously calculated across all raters for each goniometer-position-joint data set. Following the methodology for concurrent assessment of reliability [43] and previous suggestions regarding meaningful ICC values [38], the null hypothesis was that the ICCs were less than or equal to 0.75, and the alternative hypothesis was that the ICCs would be more than 0.75. The null hypothesis was considered rejected, if the LLs of 95% one-sided L-L CI for the ICCs were less than or equal to 0.75. Computation algorithms for concurrent assessment of reliability are presented in the Additional file 5.

For further reflection of intra-goniometer (i.e., intra-rater ) reliability, proportions of clinically non-meaningful ≤ 5-degree differences between the measurements obtained with the same tool in the 2 trials were calculated for each rater. Similarly, for the assessment of inter-goniometer reliability, proportions of ≤ 5-degree differences between measurements of the same rater with different instruments within the same trial were found. The observed proportions of the ≤ 5-degree differences were tested against proportion of 0.95 for statistical significance by one sample binomial tests. The reference value was estimated by calculating the LL of 99% CI for population proportion [49] using the largest previously employed sample sizes reaching 60 [32] and a generous assumption that the earlier sample proportion of ≤5-degree measurement differences was 0.99. Counts of the raters who passed the binomial tests were obtained for intuitive comparison. To assess the inter-goniometer ≤ 5-degree agreement, only the raters who passed the binomial test in both trials were included. Additionally, the best raters were selected by matching the individual successful raters across the three ≤ 5-degree agreement subgroups (i.e., across the inter-goniometer and the two intra-goniometer subgroups).

#### Analysis of the study parts B

To find whether the try-angle guides significantly changed the observed angles of the PIP joint, multiple Wilcoxon signed-rank tests with Bonferroni correction were performed for each rater-instrument-position-subposition data set in respect to the baseline joint angles obtained by using the smallest standard angles. Then the standard differences between the angles of the appropriate try-angles (i.e., between the standard angles) were calculated in respect of the smallest standard angles. Next, the lowest significant standard differences were found between the smallest standard angles and the angles of the try-angles, application of which produced significant changes in the observed PIP joint angles (Additional file 6). The lowest significant standard differences were compared with each other and with the corresponding values of the MDC derived from the SEMs of the study parts A.

#### Analysis of reliability of the diagram evaluation

Intra-rater (inter-trial) and inter-rater (intra-trial) reliability of the computerized measurements of diagrams was assessed by calculating mean differences between the appropriate pairs of measurements and their standard deviations.

## Results

### Results of exploratory data analysis

**Figure 3:**
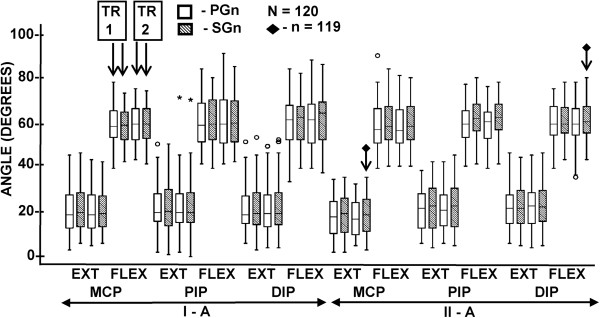
**Box plots of the joint angle measurements obtained in the study parts A.** TR = trial; PGn = paper strip goniometer; SGn = standard finger goniometer; MCP = metacarpophalangeal joint; PIP = proximal interphalangeal joint; DIP = distal interphalangeal joint; EXT = position of imitated extension; FLEX = position of imitated flexion.

The data available for the analysis included 5758 measurements from the study parts A and 1152 measurements from the study parts B. Additional file 7 presents the raw data of the study to enable rerun of the analysis and thus facilitate interpretation of the findings obtained by the uncommon statistical approaches. The descriptive statistics of the data is reflected in Additional file 8. In the study parts A, the data arranged in trial-joint-position-goniometer sets included 10 outliers with standard scores above 3.0 (Figure 3). The outliers were retained for the analysis to preserve sufficient sample size. In the study part II-A, one rater failed to perform 2 measurements with the standard goniometer, which necessitated sample size reduction of the appropriate subgroups. Normality of distribution could be assumed for almost 97% of the data sets of the study parts A arranged by the raters’ individual measurements. Although larger data aggregates failed Shapiro-Wilk test, normality could be assumed by analyzing the appropriate histograms and Q-Q plots. Therefore, having confirmation of homogeneity of the appropriate variances by Leven’s test, the analysis was continued with parametric tests based on robust ANOVA [50]. In the study parts B, Shapiro-Wilk test confirmed normal distribution in up to 90% of the sets of the differences between the appropriate subgroups of measurements.

### Results of the study parts A

The repeated measures 2x2x10 ANOVAs revealed that the main effect of goniometer was insignificant. The main effect of trial was significant for the MCP joint in imitated extension in study part I-A and in all study part II-A subgroups. The main effect of rater was significant for the MCP joint in study part I-A and in all study part II-A subgroups. Trial by rater interaction effect was observed in all the subgroups except for that of the DIP joint in position of imitated flexion. Goniometer by trial interaction was observed only in the DIP joint extension subgroup of the study part II-A. The 2x10 ANOVAs showed that goniometer and rater effects were insignificant in approximately half of the trial-position-joint data sets. Most of the two-way ANOVAs resulted in significant goniometer by rater interaction. Insignificance of all effects was observed only in the study part I-A, for the first trial measurements of the DIP joint and for the second trial measurements of the PIP joint in flexion.

Concurrent assessment of intra-rater and inter-rater reliability showed that both methods have similar reliability parameters, which, however, tended to be slightly higher for the standard goniometer (Table 1). In the statistical hypothesis testing, most of the LLs of 95% one-sided L-L CIs for the ICCs were above 0.75. In 5 out of 8 instances where the paper goniometer failed the test, the standard goniometer performed similarly. In the other three cases of failure to reject the null hypothesis for paper goniometer, the LLs of 95% one-sided L-L CIs were above 0.7. All ICCs and SEMs for the MCP joint tended to be superior to the corresponding estimates for the interphalangeal joints. Intra-rater ICCs and SEMs were higher than corresponding inter-rater reliability measures.

**Table 1:** Reliability estimates obtained in the study parts A

***Position, Study part, Characteristics, Goniometer***	***ICC (LL of 95 % one-sided L-L CI)***			***SEM in degrees***		
	**MCP**	**PIP**	**DIP**	**MCP**	**PIP**	**DIP**
EXT, I-A, Intra-R, PGn	0.88 (0.87)	0.84 (0.82)	0.89 (0.88)	3.2	4.1	3.5
SGn	0.89 (0.885)	0.86 (0.84)	0.91 (0.90)	3.1	4.2	3.3
II-A, Intra-R, PGn	0.90 (0.89)	0.86 (0.85)	0.85 (0.83)	2.8	3.3	3.8
SGn	0.89 (0.88)	0.90 (0.89)	0.87 (0.865)	2.9	3.3	3.6
I-A, Inter-R, PGn	0.87 (0.78)	0.78 (0.65)*	0.85 (0.76)	3.3	4.8	4.1
SGn	0.86 (0.77)	0.80 (0.69)*	0.88 (0.79)	3.5	4.9	3.8
II-A, Inter-R, PGn	0.86 (0.77)	0.83 (0.72)*	0.82 (0.71)*	3.2	3.8	4.1
SGn	0.87 (0.78)	0.84 (0.74)*	0.86 (0.77)	3.2	4.0	3.8
FLEX. I-A, Intra-R, PGn	0.89 (0.88)	0.86 (0.85)	0.83 (0.81)	2.8	4.2	4.3
SGn	0.91 (0.90)	0.89 (0.88)	0.86 (0.85)	2.4	3.6	3.8
II-A, Intra-R, PGn	0.90 (0.89)	0.85 (0.82)	0.82 (0.77)	3.2	3.4	3.8
SGn	0.93 (0.92)	0.87 (0.85)	0.85 (0.82)	2.8	3.2	3.4
I-A, Inter-R, PGn	0.87 (0.78)	0.83 (0.73)*	0.78 (0.66)*	3.1	4.6	4.9
SGn	0.86 (0.76)	0.86 (0.76)	0.83 (0.73)*	3.0	4.1	4.2
II-A, Inter-R, PGn	0.83 (0.72)*	0.76 (0.62)*	0.69 (0.54)*	4.2	4.3	4.9
SGn	0.88 (0.80)	0.80 (0.67)*	0.75 (0.61)*	3.5	3.9	4.4

*ICC* = intraclass correlation coefficient; *LL* = lower limit; *CI* = confidence interval; *SEM* = standard error of measurement; EXT, *FLEX* = position of imitated incomplete extension and flexion, respectively; *R* = rater; *PGn* = paper goniometer; *SGn* = standard finger goniometer; * null hypothesis retained.

**Figure 4:**
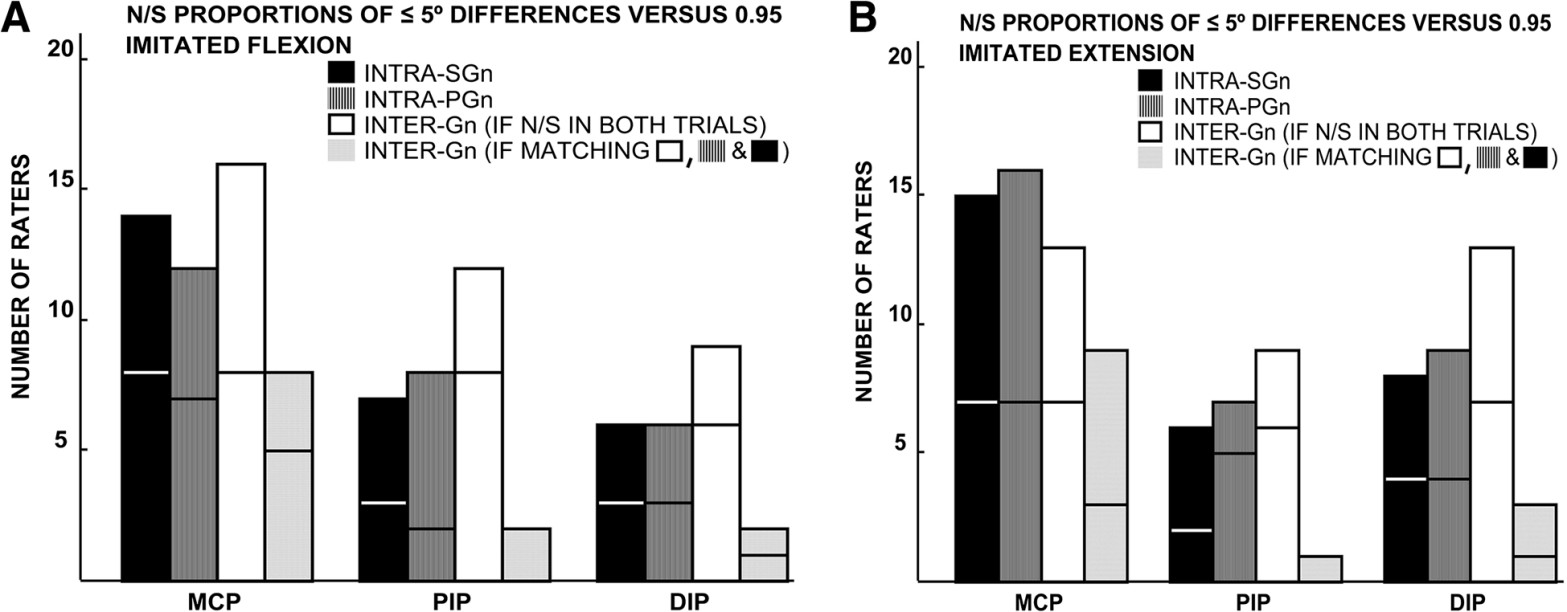
**(A, B) Summary of one sample binomial tests for the proportions of measurement differences of ≤ 5°.** N/S = not significant; SGn = standard finger goniometer; PGn = paper strip goniometer; Gn = goniometer. The parts of the bars below the horizontal lines represent stage I of the study.

The results of the binomial tests for significance of observed proportions of the clinically non-meaningful differences of ≤ 5 degrees are illustrated in Figure 4. The number of raters whose repeated measurements fell within ≤ 5 degrees of each other in proportions comparable with the criterion value of 0.95 was similar for both tools. In all joint and position subgroups except for that of MCP extension, slightly more raters passed the inter-goniometer than the intra-goniometer ≤ 5-degree agreement test. The relative increase in the number of raters who passed the binomial test for the inter-goniometer ≤ 5-degree agreement was due to the instances where the individual intra-goniometer inter-trial differences exceeded 5 degrees for both instruments, but the inter-goniometer intra-trial differences of the same measurements were within 5 degrees. Very few raters passed the binominal tests for both the intra-goniometer and inter-goniometer ≤ 5-degree agreement. The results of the binomial tests also showed that the MCP joints were evaluated more precisely than the interphalangeal articulations.

The results of the study part II-A tended to be slightly worse than those of the study part I-A.

### Results of the study parts B

According to the multiple paired Wilcoxon tests, a significant change in the PIP joint angle was mostly observed after application of the try-angles differing from the baseline angle at least by 9 degrees (Table 2). The lowest standard significant differences were similar for both goniometers and raters. The obtained lowest standard significant differences were comparable to the corresponding MDCs from the study parts A.

**Table 2:** Comparison of the minimal detectable changes with the lowest standard significant differences*

***Position, Goniometer***	***MDC***		***Lowest significant standard difference (in degrees)***			
	**I-A**	**II-A**	**Rater 1 (I-B)**	**Rater 2 (I-B)**	**Rater 3 (II-B)**	**Rater 4 (II-Bcpa)**
EXT, PGn	11.5	9.3	14	9	9	9
SGn	11.6	9.0	9	9	9	9
FLEX, PGn	9.5	11.5	9	9	9	9
SGn	9.9	8.7	13	9	9	5

*MDC* = minimal detectable changes obtained from the corresponding SEMs in Table 1; I-A, II-A, I-B, and II-B = appropriate study parts; *EXT, FLEX* = position of imitated incomplete extension and flexion, respectively; *PGn* = paper goniometer; *SGn* = standard finger goniometer; * the lowest difference between the angles of the try-angles application of which resulted in significant differences between the corresponding observed PIP joint angles according to Wilcoxon signed ranks test (see Additional file 6 for details).

### Reliability of the diagram evaluation

The mean intra-rater (inter-trial) and inter-rater (intra-trial) differences of the computerized measurements of the joint angle diagrams ranged from - 0.1 to 0.21 degrees. The mean absolute differences did not exceed 0.4 degrees. The standard deviations of the mean differences were below 0.7 degrees. All measurement differences were within 1 degree except for one occasion for each of the invited raters, where their trial-to-trial measurements disagreed by 2 degrees.

## Discussion

In the current study, reliability of diagrammatic and standard finger goniometry was assessed by employing a repeated measures design with replication, in which non-professional participants acted as raters and subjects. The diagrams of the joint angles were converted to numerical values by computerized angle measurements. The measurement errors due to the conversion were below 0.7 degrees, which is not substantial in terms of the clinically acceptable 5-degree error.

The results of all the analytical approaches support the suggestion that both goniometers can be used interchangeably. Significance of goniometer effect apparent from some of the 2x10 ANOVAs should be interpreted in conjunction with significant goniometer by rater interaction, indicating that the performance of the instrument tended to be depended on which the rater was using it. The small magnitudes of the differences between the reliability estimates of the techniques were not convincing enough to state disparity of the methods. In the three cases of failure to reject the null hypothesis for paper goniometer alone, the LLs of 95% one-sided L-L CIs levels above 0.7 can still be considered as an acceptable level of reliability for non-professional novice raters. Interchangeability of goniometers was also demonstrated by the binomial tests, which involved assessment of the inter-goniometer ≤ 5-degree agreement. It is notable, that the results of the proportion analysis echo the outcomes of parametric assessments indicating that the measurement consistency was rater and joint dependent. Parity of the goniometers was further shown by the results of the study parts B, indicating that data collected with both instruments can be similarly interpreted in an exploration of simulated change in joint range of motion over time. Decrease in the reliability estimates in the second stage of the study part A may be due to the weariness of the participants.

Straightforward comparison of the obtained results with those of the other explorations is complicated, as reliability studies differ in technical and statistical aspects [39]. Some methodological issues of the earlier studies of finger goniometry were addressed in the rationale of the study design. A more detailed reflection of the design diversity and results of the previous explorations is given in Additional file 9. Most of the intra-rater and inter-rater ICCs obtained in the current study were above 0.8, which indicates reliability [38] comparable with the previously reported values [6,10,11,17-20,25,27,29,33,37]. Most of the SEMs obtained in the current study are also in comparison with the corresponding estimates reported by the earlier researchers [9,29,33,37]. The SEM exceeding 1.8 degrees, however, indicates that the repeatability coefficient (or MDC) is above the conventional 5-degree limit. The other finger goniometric studies [2,13,15,19,23,26] have also observed intra-rater or inter-rater repeatability of more than 5 degrees.

The finding of this study that the measurements of the distal interphalangeal joint are relatively less consistent corresponds to the results of the earlier research [2,26,33,37]. This phenomenon may be associated with the stabilization difficulty of the less powered interphalangeal joints and limited phalangeal length available for the alignment of the arms of goniometers. The results of the current study also corroborate the observations of the other researchers that intra-rater reliability is better than inter-rater reliability [2,6,7,23,25,26,28,33].

The limitations of this exploration include too small sample size for the concurrent assessment of inter-goniometer reliability. This shortcoming was partly compensated by the proportion analysis of the inter-goniometer ≤ 5-degree differences. Performing the procedures in open stations may be regarded as a violation of independence of measurements, which, however, is unlikely to be substantial considering the study design features listed in the related section above.

## Conclusions

It can be concluded that that the paper goniometer and the standard goniometer can be used interchangeably by non-professional raters for the evaluation of normal finger joints. The obtained results warrant further research to assess clinical performance of the paper strip technique.

## Abbreviations

PIP: Proximal interphalangeal; MCP: Metacarpophalangeal; DIP: Distal interphalangeal; ICC: Intraclass correlation coefficient; SEM: Standard error of measurement; MDC: Minimal detectable change; ANOVA: Analysis of variance; LLs: Lower limits; L-L: Lower-limit; CI: Confidence intervals; TR: Trial; PGn: Paper strip goniometer; SGn: Standard finger goniometer; N: Number of measurements across all raters and subjects; EXT: Position of imitated incomplete extension; FLEX: Position of imitated incomplete flexion; R: Rater.

## Competing interests

The author has no competing interests to declare.

## Authors’ contributions

VM: concept, design, acquisition of data, analysis and interpretation of data, manuscript preparation and critical revisons. The author read and approved the final manuscript.

## Pre-publication history

The pre-publication history for this paper can be accessed here:

http://www.biomedcentral.com/1471-2474/14/17/prepub

## Supplementary Material

Additional file 1 **An advantage of paper strip technique over standard goniometry.** This additional file includes Figure A showing situation when proper alignment of the standard finger goniometer is impossible and Figure B demonstrating solution of the problem by means of the paper strip technique. Click here for file

Additional file 2 **Data collection design.** This additional file reflects the key features of the study design and arrangement of the try-angles in the sets. Click here for file

Additional file 3 **Standard angles.** This additional file includes angles of the try-angles and calculation of the standard differences. Click here for file

Additional file 4 **Algorithm for sample size calculation.** This additional file includes a calculation algorithm based on the formula described by Walter at al. [48]. Click here for file

Additional file 5 **Algorithms for concurrent assessment of intra-rater and inter-rater reliability.** This additional file contains the following worksheets. *Concurrent assessm algorithm Fx*. This worksheet includes an algorithm for calculation of inter-rater and intra-rater ICCs and SEMs for the case of fixed rater effects using the formulae described by Eliasziw et al. [43]; *Concurrent assessm algorithm R.* This worksheet includes an algorithm for calculation of inter-rater and intra-rater ICCs and SEMs for the case of random rater effects using the formulae described by Eliasziw et al. [43]. Click here for file

Additional file 6 Scheme of obtaining significant standard differences in the study parts B. Click here for file

Additional file 7 **Raw data of the study.** This additional file contains the following worksheets. *Data IA,IIA*. This worksheet includes a condensed version of raw data of the study parts A, appropriate measurement differences, and their dichotomized scores; *Data I-B, II-B.* This worksheet includes a condensed version of raw data of the study parts B. Click here for file

Additional file 8 **Summary of the descriptive statistics.** Includes a summary table of the essential descriptive statistics of both the study parts. Click here for file

Additional file 9 **Comparison of earlier reliability studies of standard finger goniometry.** This file includes a table with the essential results and methodological aspects of the earlier pertinent studies. Click here for file
