# Supplementary figures and images for: Reliability of the standard goniometry and diagrammatic recording of finger joint angles: a comparative study with healthy subjects and non-professional raters
Source: BMC Musculoskelet Disord. 2013 Jan 9;14:17. doi: 10.1186/1471-2474-14-17 (PMC3557198; doi:10.1186/1471-2474-14-17)

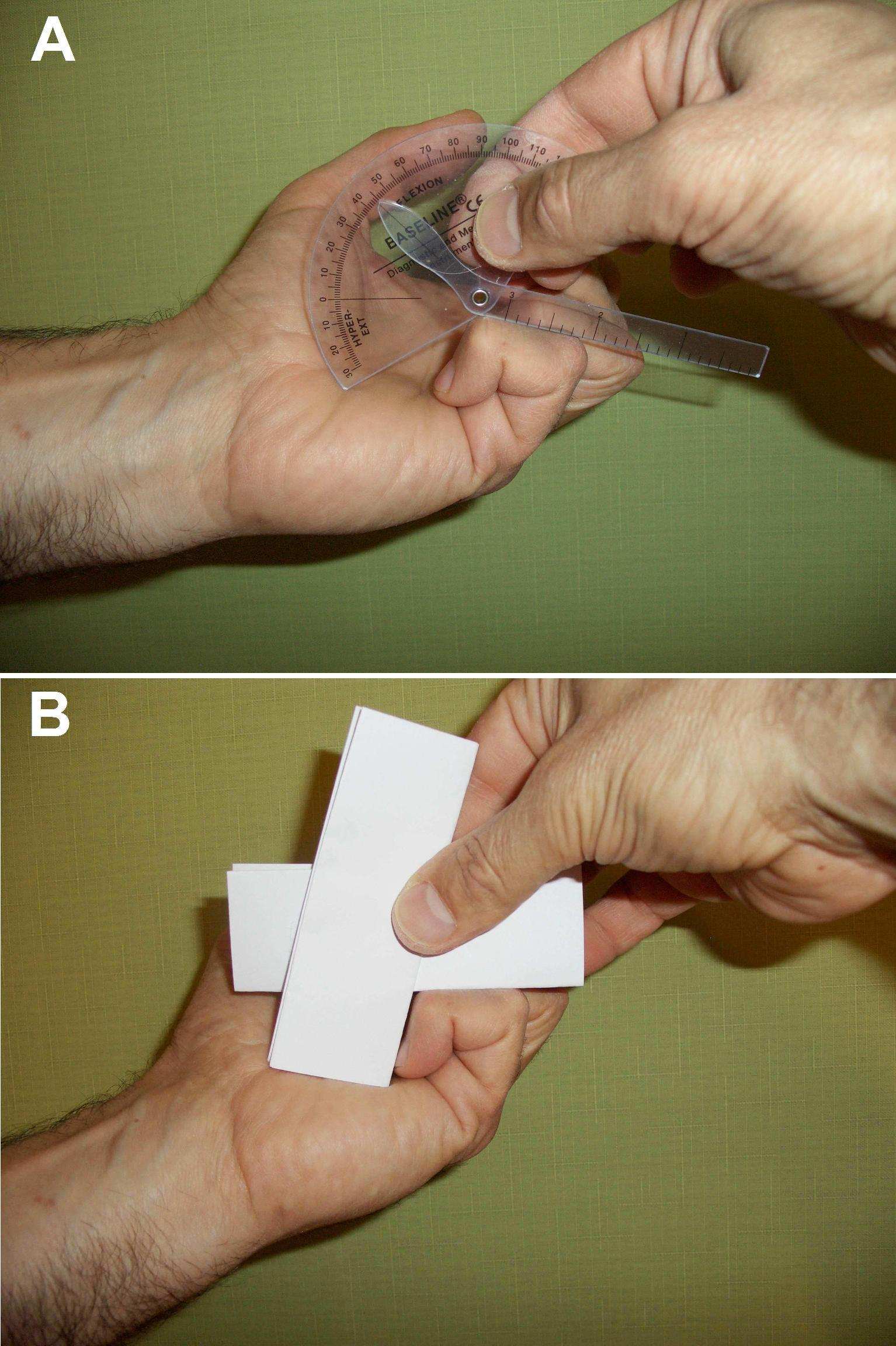

Supplement: Additional file 1 — An advantage of paper strip technique over standard goniometry. This additional file includes Figure A showing situation when proper alignment of the standard finger goniometer is impossible and Figure B demonstrating solution of the problem by means of the paper strip technique. [file 1471-2474-14-17-S1.jpeg]
