# Supplementary material for: Reliability of the standard goniometry and diagrammatic recording of finger joint angles: a comparative study with healthy subjects and non-professional raters
Source: BMC Musculoskelet Disord. 2013 Jan 9;14:17. doi: 10.1186/1471-2474-14-17 (PMC3557198; doi:10.1186/1471-2474-14-17)
Supplement: Additional file 2 — Data collection design. This additional file reflects the key features of the study design and arrangement of the try-angles in the sets. [file 1471-2474-14-17-S2.pdf]

DATA COLLECTION DESIGN

STUDY PARTS I - A & II - A

Subject (Try-angle set)	Characteristic of Individual Try-angles (Standard angles[a] by Joint - Position - Subposition)	Raters' measurements[b]								
		Rater 1				. . .	Rater 10			
		PGn[c]		SGn		. . .	PGn		SGn	
		T1	T2	T1	T2	. . .	T1	T2	T1	T2
Subject 1	MCP - FLEX - 1	[d]				. . .				
	PIP - FLEX - 1									
	DIP - FLEX - 1					. . .				
	MCP - EXT - 1									
	PIP - EXT - 1					. . .				
	DIP - EXT - 1									
.	. . .					. . .				
.	. . .					. . .				
.	. . .					. . .				
Subject 12	MCP - FLEX - 12					. . .				
	PIP - FLEX - 12									
	DIP - FLEX - 12					. . .				
	MCP - EXT - 12									
	PIP - EXT - 12					. . .				
	DIP - EXT - 12									

STUDY PARTS I - B & II - B

Rater (Try-angle set)	Characteristic of Shared Try-angles (Standard angles[a] by Joint - Position - Rater's # - Subposition)	Raters' measurements[b]				
		Subject 1		. . .	Subject 12	
		PGn[c]	SGn	. . .	PGn	SGn
Rater 11	PIP - EXT - 11 - 1	[d]				
	PIP - FLEX - 11 - 1					
	PIP - EXT - 11 - 2					
	PIP - FLEX - 11 - 2					
	PIP - EXT - 11 - 3			. . .		
	PIP - FLEX - 11 - 3					
	PIP - EXT - 11 - 4			. . .		
	PIP - FLEX - 11 - 4			. . .		
	PIP - EXT - 11 - 5					
	PIP - FLEX - 11 - 5					
	PIP - EXT - 11 - 6					
	PIP - FLEX - 11 - 6					
Rater 12	PIP - EXT - 12 - 1					
	PIP - FLEX - 12 - 1					
	PIP - EXT - 12 - 2					
	PIP - FLEX - 12 - 2					
	PIP - EXT - 12 - 3			. . .		
	PIP - FLEX - 12 - 3					
	PIP - EXT - 12 - 4			. . .		
	PIP - FLEX - 12 - 4			. . .		
	PIP - EXT - 12 - 5					
	PIP - FLEX - 12 - 5					
	PIP - EXT - 12 - 6					
	PIP - FLEX - 12 - 6					

I & II = replicate study stages; SGn = standard finger goniometer; PGn = paper strip goniometer; T = trial; MCP, PIP, and DIP = metacarpophalangeal, proximal interphalangeal, and distal interphalangeal joints, respectively; FLEX, EXT = positions of imitated flexion and extension, respectively; # = number.
[a] more details, including values of the standard angles, are presented in Additional file 3;
[b] observed angles of the joints positioned by the appropriate try-angles; [c] angle values were obtained after computerized measurements of diagrams; [d] empty cells for appropriate angles in degrees.

Note = the raters of the study stage I became subjects of the study stage II; and the subjects of the study stage I became raters of the study stage II.
