# Supplementary material for: Reliability of the standard goniometry and diagrammatic recording of finger joint angles: a comparative study with healthy subjects and non-professional raters
Source: BMC Musculoskelet Disord. 2013 Jan 9;14:17. doi: 10.1186/1471-2474-14-17 (PMC3557198; doi:10.1186/1471-2474-14-17)

V.Macionis. Reliability of the standard goniometry and diagrammatic recording of finger joint angles: a comparative study with healthy subjects and non-professional raters. Additional file 6

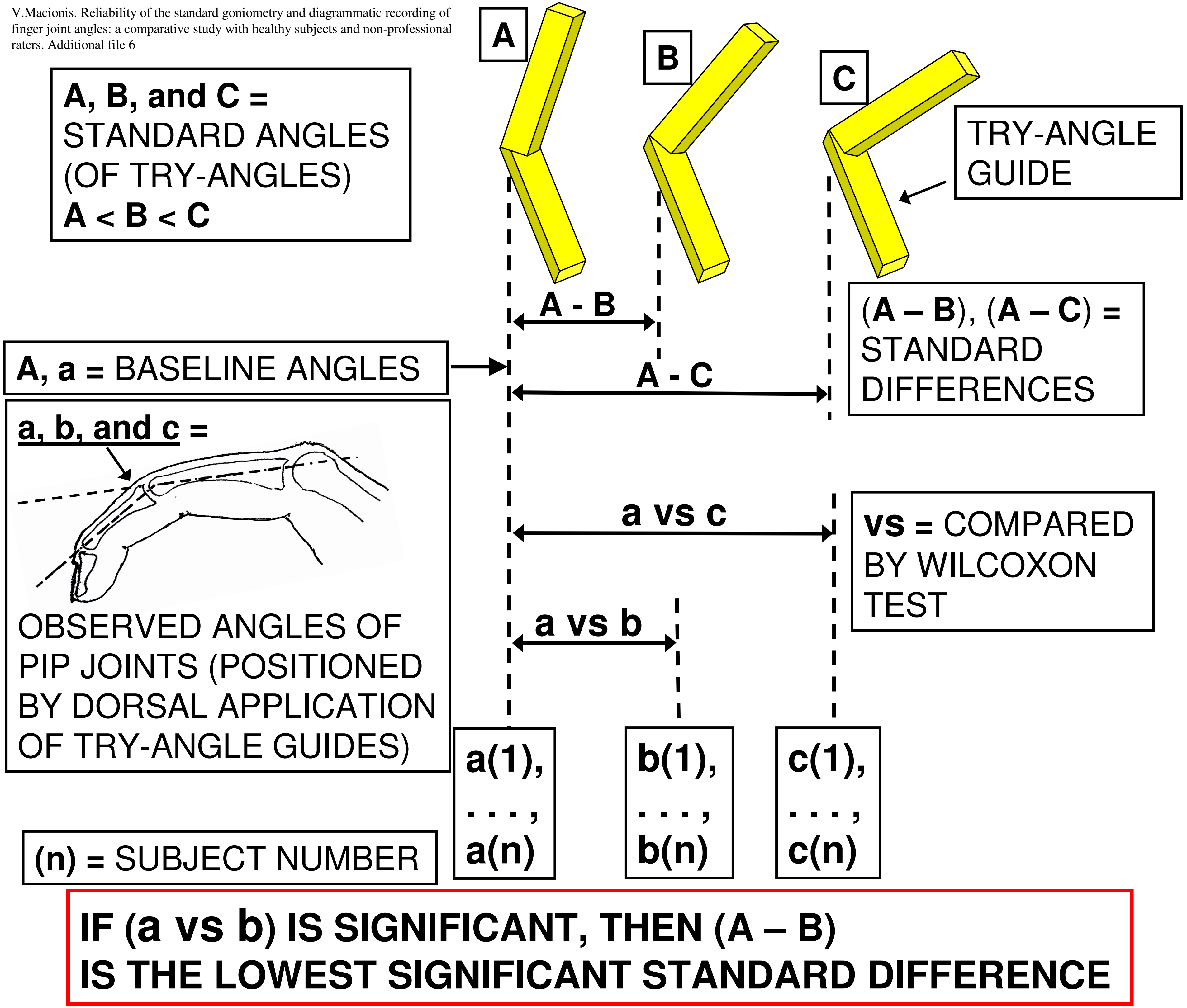

Supplement: Additional file 6 — Scheme of obtaining significant standard differences in the study parts B. [file 1471-2474-14-17-S6.pdf]
