# Supplementary material for: Reliability of the standard goniometry and diagrammatic recording of finger joint angles: a comparative study with healthy subjects and non-professional raters
Source: BMC Musculoskelet Disord. 2013 Jan 9;14:17. doi: 10.1186/1471-2474-14-17 (PMC3557198; doi:10.1186/1471-2474-14-17)
Supplement: Additional file 9 — Comparison of earlier reliability studies of standard finger goniometry. This file includes a table with the essential results and methodological aspects of the earlier pertinent studies. [file 1471-2474-14-17-S9.doc]

**Additional table Comparison of statistical and technical aspects of earlier reliability studies of standard finger goniometry (only studies presenting reliability or agreement estimates for standard goniometry are included)**

| [Reference number] Author, year of publication | # of subjects x  # of fingers (joints) | # of raters | # of trials | Breaks | Evaluated joints | Motion or position | Reliability estimates | Condition of the hand under evaluation | Graduation of goniometer |
| --- | --- | --- | --- | --- | --- | --- | --- | --- | --- |
| [6] Hamilton and Lachenbruch, 1969 | 1 x 4 | 7 | 4 | 1 d. | M, P, D | Static position | σ^2^*intra* (0.576° - 4.211°) | Normal | n/a |
| [7] Giudice, 1990 | 5 x ≥ 10 | 1 | 2 | short* |  | Passive extension (for torque range of motion) | Pearsons *r* *intra* (0.92) | Normal | n/a |
| [8] Breger-Lee D et al., 1993 | 14 x 56 | 3 | 2 | 1 d. | M | Passive extension (for torque range of motion) | ICC*intra* (0.34 - 0.83)  ICC*inter* (0.29 - 0.55) | Normal | n/a |
| [9] Dijkstra et al., 1994 | 30 x 2 | 3 | 2 | 25 min. | M | Passive hyperextension | SEM*intra* **** (2.12° - 3.88°)  SEM*inter*** (3.39° - 4.38°) | Normal | n/a |
| [10] Weiss et al., 1994 | 8 x 1 | 1 | 2 | 2 wks | M, P, D | Static position | ICC*intra* (0.98) | Normal | n/a |

**Additional table (continuation)**

| [Reference number] Author, year of publication | # of subjects x  # of fingers (joints) | # of raters | # of trials | Breaks | Evaluated joints | Motion or position | Reliability estimates | Condition of the hand under evaluation | Graduation of goniometer |
| --- | --- | --- | --- | --- | --- | --- | --- | --- | --- |
| [11] Flowers and LaStayo, 1994 | 7 x 20 | 1 | 2 | 2 - 8 wks | P | Passive extension (for torque range of motion) | ICC*intra* 0.98 | Fused joints | n/a |
| [2] Ellis et al., 1997 | 1 x 1 | 40 | 3 | short* | M, P, D | Static position  (flexion and extension) | MDC*intra* (3.8° - 6.4°)  MDC*inter* (4.4° - 9.9°) | Normal | 1° |
| [13] Goldsmith and Juzl, 1998 | 12 x n/a | 2 | n/a | n/a | n/a | n/a | MDC*inter* (7°) | n/a | n/a |
| [15] Bruton et al.,  1999 | 1 x 1 | 40 | 1 | short* | M | Static position  (flexion and extension) | MDC*inter* (4.4° - 5.9°) | Normal | 1° |
| [17] Brown et al.,  2000 | 30 x 2 | 3 | 3 | short* | TAM | Active flexion and extension | ICC*intra* (0.97 - 0.98)  ICC*inter* (0.97) | Posttraumatic conditions | 5° |

**Additional table (continuation)**

| [Reference number] Author, year of publication | # of subjects x  # of fingers (joints) | # of raters | # of trials | Breaks | Evaluated joints | Motion or position | Reliability estimates | Condition of the hand under evaluation | Graduation of goniometer |
| --- | --- | --- | --- | --- | --- | --- | --- | --- | --- |
| [18] Catalano 3rd et al., 2001 | 17 x 24 | 3-4 | n/a | n/a | TAM | Active flexion and extension | ICC*inter* (0.884 - 0.903) | Following flexor tendon injuries | n/a |
| [19] Groth et al., 2001 | 1 x 2 | 39 and 6 (ICC) | 2 | short* | P, D | Active flexion and extension | Standard deviations  (3.1° - 7.4°)  ICC*inter* (0.86 - 0.99) | Posttraumatic conditions | 1° and  5° |
| [20] Lefevre-Colau et al., 2001 | 30 x 1 | 2 | 2 | 1 hour | M, P | Passive flexion and extension | ICC*intra* (0.91 - 0.96)  ICC*inter* (0.61 - 0.79) | Rheumatoid arthritis | n/a |
| [23] Ellis and Bruton,  2002 | 1 x 3 | 51 | 3 | short* | TAM | Static position | MDC*intra* (4° - 5°)  MDC*inter* (7° - 9°) | Normal | 1° |

**Additional table (continuation)**

| [Reference number] Author, year of publication | # of subjects x  # of fingers (joints) | # of raters | # of trials | Breaks | Evaluated joints | Motion or position | Reliability estimates | Condition of the hand under evaluation | Graduation of goniometer |
| --- | --- | --- | --- | --- | --- | --- | --- | --- | --- |
| [25] Glasgow et al., 2003 | 32 x 1  (1 joint) | 2 | 2 | 5 min., short* | M, P, D | Passive flexion or extension (for torque range of motion) | ICC*inter* (0.9928)  ICC*intra* (0.9932; 0.9976)  ICC*test-retest* (0.9938; 0.9932) | Posttraumatic conditions | n/a |
| [26] Burr et al., 2003 | 1 x 2 | 40 | 3 | short* | P, D | Static position | 95th percentile of max – min  differences as *intra*  reliability (5° - 16°) | Normal | 2° and 5° |
| [27] Glasgow et al., 2004 | 24 x n/a  (38 joints) | 2 | 3 | short* | M, P | Passive flexion or extension (for torque range of motion) | Torque angle curve reliability  ICC*inter* (0.80)  ICC*intra* (0.76; 0.64) | Posttraumatic conditions | n/a |
| [28] Pratt et al., 2004 | 1 x 1 | 26 | 2 | short* | M, P, D | Active flexion | Standard deviations  (3.6° - 7.1°) | Dupuytrn‘s contracutre | 5° |

**Additional table (continuation)**

| [Reference number] Author, year of publication | # of subjects x  # of fingers (joints) | # of raters | # of trials | Breaks | Evaluated joints | Motion or position | Reliability estimates | Condition of the hand under evaluation | Graduation of goniometer |
| --- | --- | --- | --- | --- | --- | --- | --- | --- | --- |
| [29] Stam et al., 2006 | 20 x 4 | 1 | 2 | 1 week | M, P, D | Static position | ICC*intra* (0.58 - 0.83)  SEM*intra* (3.7° - 5.9°) | Normal | n/a |
| [31] Kato et al.,  2007 | 4 x 4 | 3 | 5 | short* | P | Static position | Difference between goniometric and X-ray film measurements  Means *intra* (1.5° - 2.7°)  SDs of the means (4.2° - 5.5°) | Normal (cadaver hands) | 2° and 5° |
| [33] Lewis et al.,  2010 | 20 x 1 | 7 | 3 | 5 min., 1 d., 3 wks. | M, P, D | Active and passive flexion and extension  [Data presented for flexion only] | ICC *intra* (0.43 - 0.99)  ICC*inter* (0.24 - 0.95)  MDC*intra* (0.71° - 5.54°)  CV*intra* (4% – 38%)  CV*accross all raters* (4.1% – 14.3%) | Normal | 2° |

**Additional table (continuation)**

| [Reference number] Author, year of publication | # of subjects x  # of fingers (joints) | # of raters | # of trials | Breaks | Evaluated joints | Motion or position | Reliability estimates | Condition of the hand under evaluation | Graduation of goniometer |
| --- | --- | --- | --- | --- | --- | --- | --- | --- | --- |
| [37] Engstrand et al.,  2012 | 13 x 1 | 8 | 1 | short* | M, P, D | Active flexion and extension | ICC*inter*  (0.832 - 0.973)  SEM*inter* ( 1° - 3°) | Dupuytren‘s contracture | 2° |

**Additional table (abbreviations)**

# = number; d. = day; M = metacarpophalangeal joint; P = proximal interphalangeal joint; D = distal interphalangeal joint; *intra* = intra-rater; σ^2^ = variance; n/a = not available; ICC = intraclass correlation coefficient; *inter* = inter-rater; min. = minutes; SEM = standard error of measurement; wks = weeks; MDC = minimal detectable change, method error or repeatability coefficient; TAM = total active motion; SD = standard deviation; CV = coefficient of variation; * evaluations done during the same session; ** the authors presented residual standard deviations of repeated measurements, which is the same as SEM .
